# Response prediction of radiotherapy in lung cancer patients using multimodal data

**DOI:** 10.1002/acm2.70277

**Published:** 2025-10-07

**Authors:** Xiaojuan Duan, Yushun Gong, Liang Wei, Lu Chen, Xin Song, Yongqin Li

**Affiliations:** ^1^ Department of Biomedical Engineering and Imaging Medicine Army Medical University Chongqing China; ^2^ Department of Oncology Second Affiliated Hospital of Army Medical University Chongqing China; ^3^ Department of Medical Engineering First Affiliated Hospital of Army Medical University Chongqing China

**Keywords:** Lung cancer, Multimodal, Physiological features, Response prediction

## Abstract

**Background and Purpose:**

Radiotherapy (RT) is a critical treatment for lung cancer; however, individual responses vary significantly. Pre‐treatment prediction of RT response could guide clinical decision‐making and identify patients unlikely to benefit. This study aims to predict RT response in lung cancer patients using multimodal data.

**Methods:**

Patients diagnosed with lung cancer and scheduled to undergo RT at a single institution between May 2022 and October 2024 were selected. Multimodal data, encompassing demographic, radiological, biological, and physiological characteristics, were collected 1 week before RT initiation. Treatment plans followed the International Commission on Radiation Units and Measurements (ICRU) Report 83 guidelines, developed using a commercial treatment planning system and delivered via a linear accelerator with 6 MV photon beams. Radiological and biological data were reassessed 4 weeks after completion of RT, with treatment response classified according to Response Evaluation Criteria in Solid Tumors (RECIST). The dataset was split into training (70%), validation (15%), and testing (15%) sets using a stratified random sampling approach. A back propagation neural network (BPNN) was trained on the training set, and model performance was validated on the testing set.

**Results:**

A total of 120 patients were analyzed. Of these, 41 were classified as having partial response (PR), 69 as stable disease (SD), and 10 as progressive disease (PD). Significant differences were observed among the groups in 58 characteristics, including 2 demographic, 5 radiological, 1 biological, and 50 physiological. Among the 34 features analyzed for PR prediction, the maximum vertical tumor diameter achieved an AUC of 0.699 (95% CI: 0.630–0.757). In contrast, the comprehensive BPNN model incorporating all characteristics showed an AUC of 0.855 (95% CI: 0.843‐0.875), with a prediction mean squared error (MSE) of 0.07. Similarly, among the 36 features analyzed for PD prediction, the zero‐crossing ratio of surface electromyography signals achieved an AUC of 0.750 (95% CI: 0.648–0.841). The comprehensive model further increased AUC to 0.929 (95% CI: 0.900–0.960), with a prediction MSE of 0.01.

**Conclusion:**

Pretreatment demographic, radiological, and physiological characteristics were associated with RT response in lung cancer patients. The developed BPNN models leveraging multimodal data effectively predicted PR and PD, to guide personalized treatment strategies and to identify patients unlikely to benefit from RT.

## 1 INTRODUCTION

Lung cancer is the most common malignant tumor, with an incidence rate of 52.2 per 100 000 among men and 17.8 per 100 000 among women, and an overall 5‐year survival rate of 28.7%, according to the national cancer report.^1^ Radiotherapy (RT) significantly improves survival and local control in lung cancer patients, particularly with the advent of intensity‐modulated RT from 2013 to 2020.^2^ In limited‐stage small cell lung cancer, high‐dose thoracic RT extended median overall survival by 21.2 months.^3^ Additionally, stereotactic ablative RT achieves over 70% 5‐year local control in early‐stage NSCLC.^4^

However, significant inter‐patient heterogeneity in RT response continues to pose challenges to the therapeutic landscape.^5^

Current strategies for evaluating lung cancer treatment response, including conventional anatomical imaging, functional imaging modalities, and emerging serum biomarkers, are all subject to inherent temporal delays.^6^, ^7^, ^8^ Notably, growing evidence indicates that accurate pretreatment prediction of lung cancer response could potentially reduce treatment‐related toxicity by 23%–41%.^9^, ^10^ Recent investigations have increasingly focused on multimodal data integration to enhance prediction power. Vanguri et al. developed a comprehensive framework that integrates clinical, pathological, radiological, and transcriptomic data to predict immunotherapy response in non‐small cell lung cancer (NSCLC).^11^ Meanwhile, Vaios et al. evaluated combined RT and immunotherapy in NSCLC patients with brain metastases and demonstrated the potential of machine‐learning approaches for personalized treatment optimization.^12^ Moreover, research by another group identified CXCL10, CXCL2, and CXCL14 as predictors of radiation pneumonitis in lung cancer patients receiving combined immunotherapy and RT. These findings align with ongoing research into predictive models for treatment‐related pneumonitis.^13^, ^14^, ^15^ However, current response prediction models for lung cancer remain suboptimal, with limited clinical validation, and are not yet incorporated into guideline recommendations. Consequently, more robust biomarkers are required to accurately predict responses and effectively stratify patients who will or will not benefit from therapy.

Parallel to these developments, advancements in physiological monitoring have significantly enhanced the treatment outcomes for lung cancer patients,^16^ improving the management of their physiological conditions throughout treatment, and more importantly for this study, provided a rich source of predictive data. Emerging evidence suggests that fluctuations in the autonomic nervous system (heart rate variability, HRV), respiratory patterns, and cutaneous bioimpedance changes may encode systemic responses to radiation injury and tumor cell death.^17^, ^18^ For instance, preliminary studies have shown that HRV can predict survival in cancer patients.^19^ Cotogni et al. explored the use of bioimpedance analysis to track cancer patients undergoing chemotherapy and home parenteral nutrition.^18^ Chaudhari et al. introduced non‐invasive biosensors for continuous health monitoring, emphasizing the applications and benefits of non‐invasive biological signals in tracking physiological parameters.^20^ These continuous, non‐invasive biosignals offer a level of temporal resolution unattainable through conventional imaging modalities, capturing dynamic biological processes during fractionated RT and highlighting their potential as innovative predictors.

Machine‐learning architectures offer a powerful framework for decoding intricate physiological data.^21^ However, the application of machine‐learning models leveraging multimodal data based on physiological characteristics to predict the RT response in lung cancer patients has been scarcely explored. This study aims to develop machine‐learning models that integrate demographic, radiological, biological, and physiological characteristics acquired prior to RT to predict the RT response in lung cancer patients.

## 2 METHODS

### 2.1 Study flowchart

**FIGURE 1:**
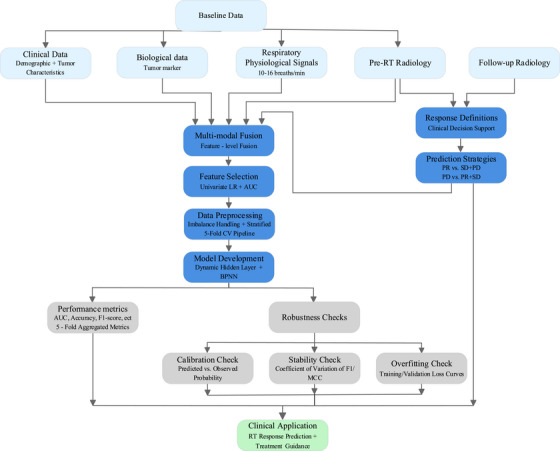
Flowchart of radiotherapy response prediction model development.

To develop the RT response prediction model, we followed a structured workflow (Figure 1) encompassing multi‐source baseline data collection (clinical, biological, physiological, radiological), sequential multi‐modal fusion, feature selection, data preprocessing (imbalance handling, stratified 5‐fold cross‐validation), model training (dynamic hidden layer + BNN), robustness validation (calibration, stability, overfitting checks), and clinical application translation, with each step visualized in the flowchart.

### 2.2 Study population

Data were collected from the department of oncology at a single institution between May 2022 and December 2024. Eligibility criteria were: (1) age 18–75 years; (2) histologically or cytologically confirmed primary lung cancer; and (3) scheduled to receive RT within the study period. Patients were excluded if they were younger than 18 or older than 75 years, or if they lacked the capacity to give informed consent or adhere to study procedures because of severe cognitive impairment or psychiatric disorders. The study protocol was approved by the Institutional Review Board of the involved institution, and all procedures adhered to the principles of the Declaration of Helsinki.

#### 2.2.1 Multi‐modal data collection and processing

Baseline assessments were conducted one week before the initiation of RT, with a median of 7 days (range: 3–10 days) prior to RT. These assessments included demographic data, clinical examination results, laboratory test results, and physiological characteristics derived from physiological signal acquisition.

#### 2.2.2 Pre‐RT multi‐modal data collection

##### Clinical and imaging data

Demographic data (age, sex, medical history, tumor subtypes, prior treatments, and other details), radiological characteristics, including the larger maximum diameter (MaxD) and maximum vertical diameter (MaxVD) of target lesions, tumor location, and stage, and biological markers (carcinoembryonic antigen [CEA], carbohydrate antigen 15‐3 [CA15‐3], carbohydrate antigen 19‐9 [CA19‐9], neuron‐specific enolase [NSE], squamous cell carcinoma antigen [SCC], cytokeratin fragment 21‐1 [Cyfra21‐1], carbohydrate antigen 125 [CA125], and gastrin‐releasing peptide precursor [ProGRP]) were extracted from the electronic medical records of the patients.

##### Physiological signal acquisition and feature extraction

Participants were informed about the study's objectives, procedures, and breathing protocols and were advised to avoid coffee, tea, alcohol, strenuous exercise, and significant mood changes on the evening before and the day of monitoring. Written informed consent was then obtained. A thermal gas mass flowmeter connected to a ventilator tube via an anesthesia mask was used to record the airflow signal. Five disposable Ag‐AgCl electrodes were placed on the participants’ skin in a bipolar configuration to capture three additional respiratory‐related signals: transthoracic impedance, surface diaphragm electromyography, and lead II electrocardiogram. After a 5‐minute rest, participants lay supine on the examination bed. While listening to yoga music and following breathing guidance, their breathing rate was increased stepwise: 10, 12, 14, and 16 breaths per minute. Under controlled experimental conditions, four signals were synchronized and recorded over a 5‐minute period. All procedures were conducted by trained personnel in a standardized manner to ensure data accuracy and reliability.

Using a four‐segment approach stratified by the above respiratory rate (10–16 breaths/min), distinct physiological characteristics were extracted from each corresponding segment. These characteristics included time‐domain features (e.g., mean and standard deviation [SD] of respiratory rate), frequency‐domain features (e.g., power spectral density), and nonlinear features (e.g., sample entropy and fractal dimension). All engineered features (time‐domain, frequency‐domain, and nonlinear) were provided in Table S6, which maps raw signals to derived features for full transparency.

#### 2.2.3 RT delivery

Patients were immobilized with a chest thermoplastic mask, and CT simulation was performed to obtain 3‐D images of the tumor and surrounding tissues. Target volumes were delineated by radiation oncologists according to ICRU Report 83 guidelines. Intensity‐modulated radiation therapy (IMRT) plans were then generated in a commercial treatment‐planning system and delivered on a 6‐MV linear accelerator.

#### 2.2.4 Post‐RT follow‐up assessment

Repeat radiological, biological, and physiological data were collected as part of the post‐treatment evaluations 4 weeks after RT completion (range 28–38 days).

### 2.3 Outcome definitions and prediction strategies

Tumor RT response, categorized according to the Response Evaluation Criteria in Solid Tumors (RECIST, v1.1), includes: (i) partial remission (PR), defined as a ≥30% reduction in tumor size compared to the baseline measurement; (ii) disease stabilization (SD), where the tumor size does not meet the criteria for either PR or disease progression; and (iii) disease progression (PD), characterized by a ≥20% increase in tumor size.

To address distinct clinical priorities, two prediction strategies were designed. Strategy I (treatment response): To predict PR, SD, and PD were grouped as a non‐response group (SD+PD) to contrast with the PR group. Strategy II (disease status): To predict PD, PR, and SD were grouped as a disease control group (PR+SD) to contrast with the PD group.

### 2.4 Data analysis and machine learning pipeline

This section details the analytical framework for processing multi‐modal data, developing predictive models, and validating their performance, with a focus on statistical rigor and model reliability.

#### 2.4.1 Statistics analysis

Categorical variables were presented as counts and percentages, while continuous variables were reported as mean ± SD for normally distributed data (assessed by the Shapiro–Wilk test) or as median (interquartile range [IQR]) for non‐normally distributed data. Homogeneity of variance was assessed using Levene's test. Statistical tests were selected based on data distribution and sample size. For categorical variables, the *χ* ^2^ test was used. For continuous variables, independent *t*‐tests or one‐way ANOVA were applied for normally distributed data, and Mann–Whitney U or Kruskal–Wallis tests were used for non‐normally distributed data.

#### 2.4.2 Machine learning pipeline

##### Data preprocessing

This step aimed to integrate heterogeneous data sources into a unified feature matrix while preserving data integrity and avoiding leakage.

###### Multi‐modal fusion

To integrate complementary information from heterogeneous data sources, we implemented a feature‐level fusion strategy involving two core steps. First, all continuous features across all modalities were standardized using *z*‐score normalization, with parameters (mean and SD) computed exclusively from the training set to prevent data leakage. Second, categorical features were one‐hot encoded to preserve their nominal properties and ensure compatibility with the standardized continuous feature matrix. The preprocessed continuous and encoded categorical features were then concatenated into a single matrix, retaining explicit modality labels to facilitate post‐hoc interpretability. This standardized approach balanced cross‐modal comparability while preserving the integrity of clinical and biological signals, enabling the model to capture meaningful interactions between tumor characteristics and patient physiological status.

###### Feature selection

Feature selection followed three sequential stages: first, candidate predictors—covering demographic, radiological, biological, and respiratory physiological signal indicators—were identified from baseline integrated datasets by screening for features with significant differences across outcome groups; second, univariate logistic regression (LR) analyses were applied to these differential features to calculate individual predictive value (quantified using the area under the curve, AUC), and features meeting the predefined efficacy threshold (AUC ≥ 0.6) were further filtered for two strategies (Strategy I: PR vs. SD+PD; Strategy II: PD vs. PR+SD); finally, an experienced radiation oncologist reviewed the filtered sets, appended clinically relevant and scenario‐appropriate variables, and the resulting feature pools were adopted for multivariate modeling.

##### Class imbalance handling

To address the pronounced class imbalance in our dataset (34% PR, 58% SD, 8% PD) while preventing overfitting and biased evaluation, we implemented a three‐pronged strategy: First, we implemented a signal‐level oversampling strategy tailored to the underrepresented PD group: for each of the 10 PD patients—each with 4 respiratory rate recordings—we extracted 5 non‐overlapping signal segments per recording to enrich the PD sample pool while preserving the integrity of the original physiological signals. In contrast, to avoid over‐representing the majority classes (PR and SD), we applied a more conservative sampling approach, extracting only 1 non‐overlapping segment per recording for the PR patients and SD patients. Second, stratified 5‐fold cross‐validation was employed to ensure consistent class distributions across training/validation splits (details in Model Training and Testing). Third, strict data leakage prevention was enforced by confining all segments from the same patient to either the training or test set during partitioning, thereby preventing information leakage across folds and maintaining evaluation rigor.

##### Model development

To evaluate the predictive value of different feature combinations, we developed and compared single‐modal and multi‐modal predictive models using a back propagation neural network (BPNN) framework, with a focus on optimizing architecture and generalizability.

To quantify the incremental value of integrating multi‐modal data, models were constructed based on distinct feature combinations: four single‐modal standalone models using individual modalities (Demographic [Demo], Radiological [Rad], Biological [Biolo], and Physiological [Physio] features) and two multi‐modal integrated models to assess cross‐modality synergies—specifically, Demo+Rad+Physio (combining demographic, radiological, and physiological data) and Demo+Rad+Biolo (combining demographic, radiological, and biological data). All models adopted the same BPNN framework, with input layer dimensions dynamically adjusted to match the feature count of each modality combination (e.g., fewer neurons for single‐modal inputs with fewer features, more neurons for multi‐modal inputs with integrated features), ensuring this standardized architectural design balanced fair comparability across models with accommodation of differences in input dimensionality.

Multimodal baseline data were processed using stratified five‐fold cross‐validation to maintain consistent class distributions across training (70%), validation (15%), and test (15%) subsets, preserving the original class distributions (PR: 34%, SD: 58%, PD: 8%) to mitigate imbalance effects. Random partitioning was applied within each stratum to ensure unbiased evaluation across folds.

The BPNN architecture with dynamically adjusted hidden layers was employed to enhance representational capacity. The number of neurons in the hidden layer was determined by evaluating configurations within the range of $\sqrt{\mathrm{input}\mathrm{nodes}+\mathrm{output}\mathrm{nodes}}\pm 10$, selecting the optimal count based on the highest training accuracy.^22^, ^23^ Early stopping (max_fail = 7) prevented overfitting by halting training if validation loss plateaued. Complete configuration details are provided in Table 1.

**TABLE 1:** BPNN architecture specifications and training parameters.

Component	Specification
Activation	Tansig (hidden), purelin (output)
Training algorithm	Levenberg–Marquardt (trainlm)
Key parameters	
Learning rate	—
Epoch	500
Error threshold	10^−6^
Regularization parameter	0.001
Batch processing	Full‐batch
Early stopping (max_fail)	3

##### Model evaluation framework

###### Metrics

To comprehensively evaluate model performance, we employed a multi‐metric framework based on cross‐validation results. The performance of the models was estimated using five iterations of cross‐validation. Reported metrics included the mean values with 95% confidence intervals (CIs) across the five cross‐validation iterations. Multiple performance metrics were used for the comparison of different models, including the area under the receiver operating characteristic curve (AUC; primary endpoint), accuracy, sensitivity, specificity, positive predictive value (PPV), and F1‐score. Additionally, we generated bar charts with 95% CIs as error bars to visually compare these metrics across modality combinations, illustrating AUC and other key metrics for single and multi‐modal models in PR and PD prediction tasks.

###### Robustness checks

**Calibration**. Model calibration was assessed by comparing observed probabilities with predicted probabilities, visualized via calibration curves, to evaluate the agreement between predicted and observed outcomes.

**Stability Across Cross‐Validation Folds**. Performance consistency across five cross‐validation folds was evaluated as follows: For the PR and PD tasks, we computed the F1‐score and Matthews Correlation Coefficient (MCC) across five stratified folds. To assess their stability, we used the coefficient of variation (CV). A CV threshold of < 15% — a commonly used cutoff for stability in imbalanced datasets—^24^ was considered evidence of stable discriminative ability.

**Overfitting Control**. To ensure model generalization and mitigate overfitting, we implemented rigorous monitoring and control strategies throughout the training process. For each fold of the five‐fold cross‐validation (both PR and PD prediction tasks), we tracked training and validation loss curves in real time to assess convergence patterns. Specifically, we recorded loss values at each epoch, with training loss reflecting the model's fit to the training data and validation loss indicating its performance on unseen validation samples. An early stopping mechanism was applied: training terminated when validation loss stabilized (no significant decrease over 5 consecutive epochs), preventing overfitting to noise. These processes allowed us to assess model convergence and validate overfitting mitigation.

## 3 RESULTS

### 3.1 Cohort characteristics

**FIGURE 2:**
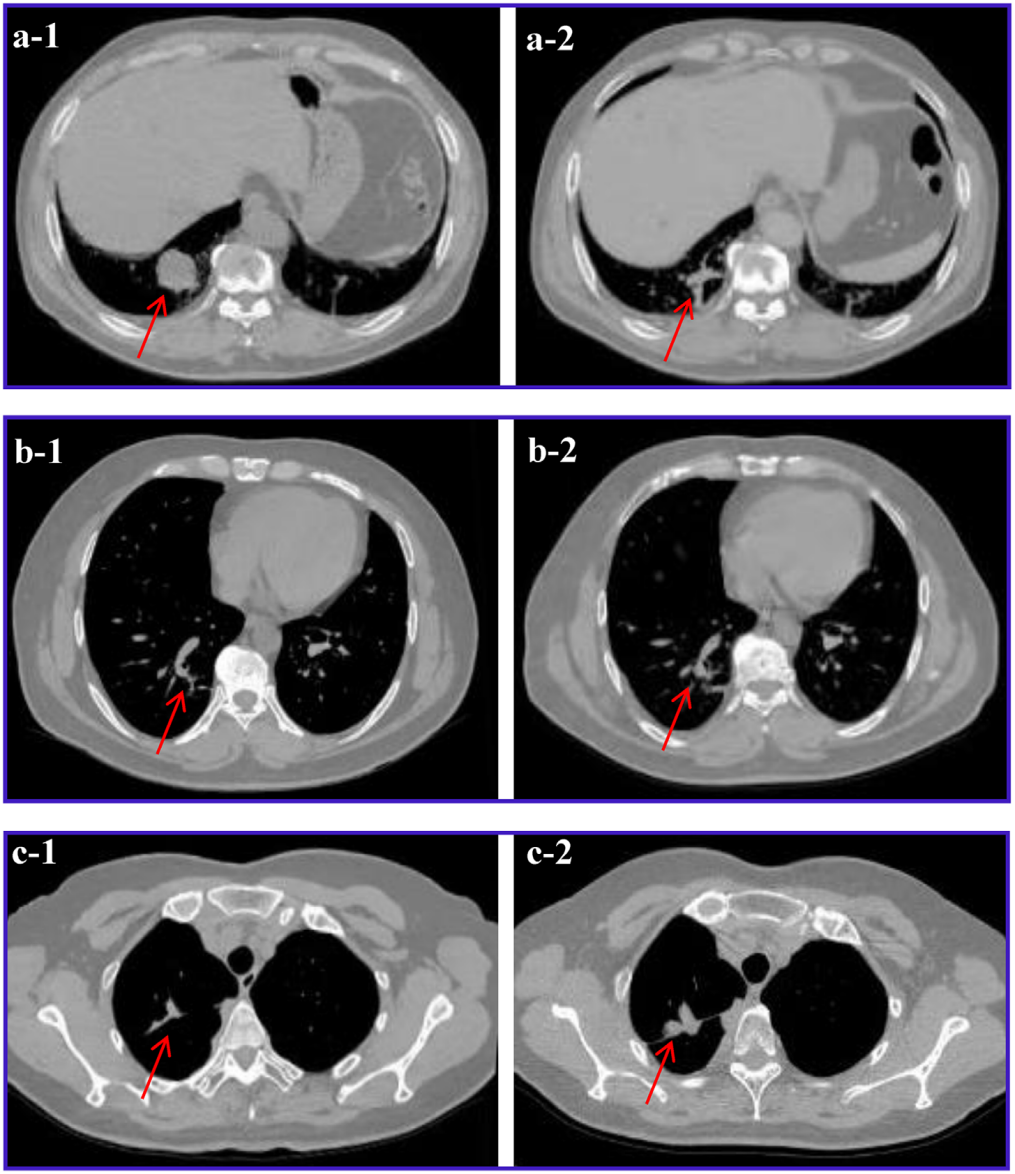
CT sections illustrating the largest tumor diameters. (a‐1) and (a‐2) depict a PR case, where the tumor size decreased from 3.9  × 3.2 cm in (a‐1) to 1.2  × 0.9 cm in (a‐2). (b‐1) and (b‐2) represent an SD case, with tumor size changing from 2.3  × 1.7 cm in (b‐1) to 2.2  × 1.7 cm in (b‐2). (c‐1) and (c‐2) show a PD case, in which the tumor size increased from 1.2  × 0.8 cm in (c‐1) to 1.3  × 1.7 cm in (c‐2).

**FIGURE 3:**
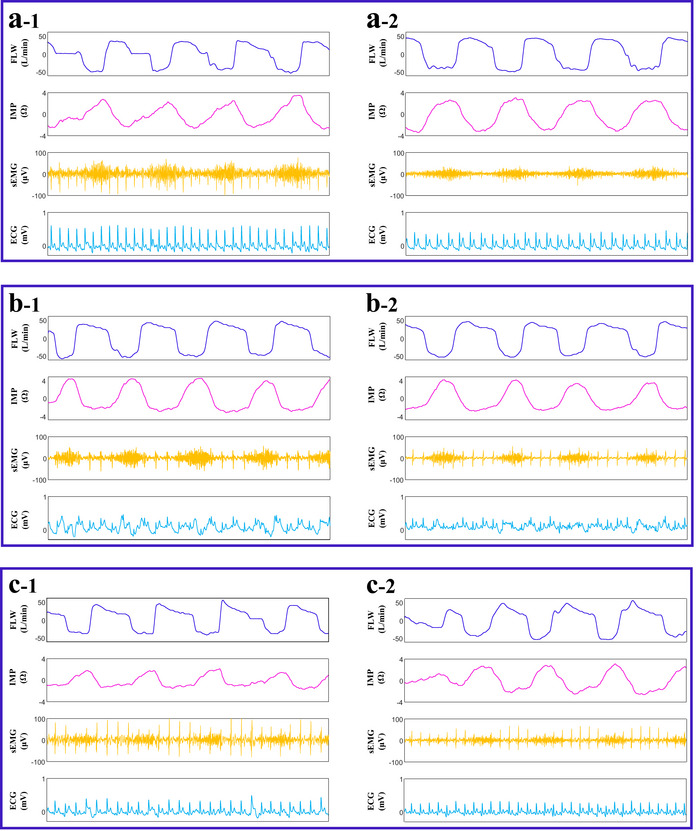
Signal recordings. (a‐1) and (a‐2) correspond to the PR case presented in Figure 1a. (b‐1) and (b‐2) pertain to the SD case shown in Figure 1b. (c‐1) and (c‐2) are associated with the PD case depicted in Figure 1c.

Among 120 patients (41 PR, 69 SD, 10 PD), baseline and 4‐week post‐RT data were analyzed to identify response predictors. Baseline distributions of sex, age, smoking status, prior therapy, tumor size, serum biomarkers, and 53 physiological variables are summarised in Tables 2, 3, 4, 5. Only prior chemotherapy and surgery rates, MaxD/MaxVD, ProGRP, NSE, and physiological indices differed significantly across response categories (all *p* < 0.05; details in Tables S1–S4). Post‐RT, larger‐nodule MaxD and MaxVD remained the only radiological features that discriminated PR from non‐PR (Table 6), while ProGRP decreased progressively from PR to PD (Table 7). Representative radiological changes and physiological signal trajectories are shown in Figures 2 and 3, respectively.

**TABLE 2:** Demographic characteristics according to the RICIST v1.

Characteristics	PR (*N* = 41)	SD (*N* = 69)	PD (*N* = 10)	*p‐value*
Male Sex, *n*(%)	38 (92.68)	65 (94.20)	8 (80.00)	0.28
Age, yr	55.20 ± 8.31	57.71 ± 7.98	61.10 ± 5.85	0.06
Height, cm	165.02 ± 6.00	165.77 ± 6.38	163.50 ± 7.06	0.53
Wight, kg	66.88 ± 9.72	65.23 ± 10.12	65.90 ± 15.74	0.73
BMI, kg/m2	25.54 ± 3.23	23.68 ± 2.99	24.51 ± 4.92	0.37
Chest, cm	94.60 ± 6.10	94.15 ± 6.11	94.25 ± 10.70	0.94
Smoking, *n*(%)	23 (56.10)	32 (46.38)	2 (20.00)	0.12
Drinking, *n*(%)	7 (17.07)	8 (11.59)	1 (10.00)	0.68
**Other completed treatments, *n*(%)**				
Surgery	0 (0.00)	1 (1.45)	2 (20.00)	0.01
Chemotherapy	39 (95.10)	67 (97.10)	7 (70.00)	0.00
Immunotherapy	14 (34.15)	23 (33.33)	1 (10.00)	0.28
Targeted therapy	2 (4.88)	11 (15.94)	3 (30.00)	0.14
**Medical condition, *n*(%)**				
Other lung disease	9 (21.95)	12 (17.39)	2 (2.00)	0.84
Cardiac disease	1 (2.44)	1 (1.45)	0 (0.00)	0.84
Hypertension	6 (14.63)	16 (23.19)	1 (10.00)	0.78
Diabetes	2 (4.88)	5 (7.25)	1 (10.00)	0.62
Medication	4 (9.76)	10 (14.49)	3 (30.00)	0.26
**Tissue type, *n*(%)**				
Adenocarcinoma	8 (19.51)	14 (20.29)	3 (30.00)	0.78
Squamous‐cell Ca.	11 (26.83)	22 (31.88)	4 (40.00)	0.74
Small cell lung Ca.	21 (51.22)	28 (40.58)	3 (30.00)	0.32
Other	1 (2.44)	5 (7.25)	0 (0.00)	0.64

Abbreviations: Ca, cancer; PD, progressive disease; PR, partial response; RECIST v1.1, response evaluation criteria in solid tumors (vision 1.1); and SD, stable disease.

**TABLE 3:** Baseline radiological characteristics according to the RICIST v1.1.

Characteristics	PR (*N* = 41)	SD (*N* = 69)	PD (*N* = 10)	*p‐value*
**Size, mm**				
MaxD‐tumor 1	3.70 (2.10)	2.90 (2.80)	1.15 (1.10)	0.00
MaxVD‐tumor 1	2.60 (2.60)	1.90 (1.70)	0.50 (1.35)	0.00
MaxD‐ tumor 2	2.65 (2.48)	2.10 (1.60)	0.60 (1.75)	0.00
MaxVD‐tumor 2	0.00 (0.00)	0.00 (0.40)	0.00 (0.00)	0.00
MaxD‐larger nodule	0.00 (0.00)	0.00 (0.30)	0.00 (0.00)	0.98
MaxVD‐larger nodule	1.00 (1.76)	1.10 (1.55)	0.60 (0.23)	0.97
**Stage, *n*(%)**				
Stage I	1 (2.44)	5 (7.25)	5 (50.00)	0.59
Stage II	3 (7.32)	3 (4.35)	0 (0.00)	0.39
Stage III	28 (62.29)	36 (52.17)	3 (30.00)	0.05
Stage IV	8 (19.51)	24 (34.78)	2 (20.00)	0.24
**Location, *n*(%)**				
Left upper lobe	11 (26.83)	20 (28.99)	5 (50.00)	0.38
Left lower lobe	6 (14.63)	11 (15.94)	1 (10.00)	0.87
Left hilum	6 (14.63)	9 (13.04)	0 (0.00)	0.38
Right upper lobe	8 (19.51)	16 (23.19)	1 (10.00)	0.64
Right middle lobe	3 (7.32)	6 (8.70)	0 (0.00)	0.61
Right lower lobe	4 (9.76)	12 (17.39)	3 (30.00)	0.37
Right hilum	4 (9.76)	8 (11.59)	0 (0.00)	0.52

Abbreviations: MaxD, maximum diameter and MaxVD, maximum vertical diameter.

**TABLE 4:** Baseline biological characteristics according to the RICIST v1.1.

Characteristics	PR (*N* = 41)	SD (*N* = 69)	PD (*N* = 10)	*p‐value*
CEA, ng/mL	2.68 (5.09)	2.44 (2.59)	2.47 (1.80)	0.48
CA15‐3, U/mL	12.50 (6.17)	11.92 (8.93)	12.50 (6.96)	0.92
CA19‐9, U/mL	8.58 (15.87)	8.79 (14.60)	5.44 (0.35)	0.53
NSE, ng/mL	13.19 (6.80)	10.49 (3.90)	8.98 (2.57)	0.07
CA15‐4, U/mL	0.90 (0.83)	0.80 (0.70)	0.70 (1.50)	0.93
Cyfra21‐1, ng/mL	2.50 (2.53)	1.78 (0.98)	1.69 (1.53)	0.13
CA125, U/mL	19.00 (23.48)	17.90 (13.13)	11.40 (7.30)	0.26
ProGRP, pg/mL	98.91 (616.28)	39.42 (32.95)	46.75 (21.61)	0.01

Abbreviations: CA125, carbohydrate antigen; CA15‐3, carbohydrate antigen; CA15‐4, squamous cell carcinoma antigen; CA19‐9, carbohydrate antigen; CEA, carcinoembryonic antigen; Cyfra21‐1, cytokeratin; NSE, neuron specific enolase; and ProGRP, gastrin‐releasing peptide precursor.

**TABLE 5:** Baseline physiological characteristics according to the RICIST v1.1.

Characteristics	PR (*N* = 41)	SD (*N* = 69)	PD (*N* = 10)	*p‐value*
**Airflow**				
Avg inhale volume, L	65.40 (23.79)	57.85 (22.98)	52.32 (21.31)	0.00
Avg exhale volume, L	64.14 (22.27)	57.54 (23.06)	53.42 (22.33)	0.00
Avg peak inpiratory flow, L/s	0.76 (0.23)	0.69 (0.21)	0.60 (0.28)	0.00
Avg peak exspiratory flow, L/s	0.63 (0.25)	0.58 (0.17)	0.56 (0.26)	0.00
Avg exhale duration, min	2.55 (0.77)	2.55 (0.66)	2.46 (0.77)	0.00
Avg tidal volume, L	129.54 (45.45)	115.39 (43.94)	105.74 (42.54)	0.00
Minute Ventilation, L	28.19 (9.10)	25.67 (7.93)	23.17 (8.77)	0.00
Duty cycle of inhale	0.43 (0.06)	0.43 (0.06)	0.46 (0.10)	0.00
Duty cycle of exhale	0.54 (0.07)	0.55 (0.08)	0.50 (0.11)	0.00
Inhale time to trough, s	0.56 (0.35)	0.65 (0.34)	0.56 (0.40)	0.29
Exhale time to peak, s	0.82 (0.41)	0.77 (0.37)	0.70 (0.43)	0.30
**Thoracic impedance**				
Respiratory rate, bpm	12.71 (3.71)	12.87 (3.07)	13.88 (4.50)	0.00
I:E ratio	0.74 (0.21)	0.74 (0.20)	0.79 (0.31)	0.00
Impedance value, Ω	1.79 (1.39)	1.34 (0.92)	1.32 (0.78)	0.00
Maximum value, Ω	2.85 (2.03)	2.05 (1.52)	2.02 (1.27)	0.00
Median value, Ω	−0.54 (0.43)	−0.32 (0.34)	−0.50 (0.40)	0.00
Minimum value, Ω	−2.72 (1.66)	−1.95 (1.26)	−2.18 (0.86)	0.00
Peak‐to‐peak value, Ω	5.35 (3.83)	4.09 (2.68)	4.14 (1.84)	0.00
Avg rectified value, Ω	1.39 (1.26)	0.99 (0.69)	1.12 (0.38)	0.00
RMS amplitude, Ω	1.36 (1.09)	0.92 (0.70)	0.99 (0.34)	0.09
Centroid frequency, Hz	0.31 (0.14)	0.35 (0.15)	0.34 (0.09)	0.00
RMS frequency, Hz	2.41 (2.46)	3.16 (1.98)	2.98 (0.78)	0.00
**sEMG**				
Mean absolute value, µV	10.42 (7.57)	9.46 (6.27)	10.67 (5.07)	0.00
Standard deviation, µV	18.81 (13.92)	15.07 (11.35)	22.92 (17.84)	0.00
Maximum amplitude, µV	112.83 (80.55)	91.95 (64.68)	99.36 (48.82)	0.00
Median amplitude, µV	0.92 (0.43)	1.35 (0.43)	1.35 (0.43)	0.00
RMS amplitude, µV	8.11 (5.28)	7.07 (4.65)	8.22 (4.34)	0.00
Zero crossing ratio	0.14 (0.04)	0.13 (0.03)	0.11 (0.02)	0.00
Slope sign change ratio	0.29 (0.07)	0.27 (0.06)	0.24 (0.06)	0.00
Willision amplitude	0.95 (0.04)	0.94 (0.05)	0.93 (0.08)	0.00
Logarithmic detection, µV	5.21 (3.71)	4.84 (3.21)	5.97 (2.20)	0.00
Integrated value	10.42 (7.57)	9.46 (6.86)	10.72 (5.11)	0.00
Autoregressive coefficients	−0.15 (0.06)	−0.16 (0.08)	−0.17 (0.11)	0.00
Median frequency, Hz	29.72 (8.49)	31.22 (6.49)	37.22 (8.31)	0.00
RMS frequency, Hz	29.98 (16.33)	31.42 (11.55)	36.06 (10.69)	0.00
**HRV**				
CPC	0.56 (0.24)	0.56 (0.22)	0.54 (0.24)	0.04
Heart rate, bpm	762.33 (154.69)	743.32 (134.09)	747.98 (112.43)	0.00
AVNN, ms	760.00 (175.00)	735.00 (149.00)	748.00 (113.00)	0.00
Median NN, ms	17.13 (13.60)	15.51 (15.50)	14.38 (11.10)	0.00
SDNN, ms	49.00 (40.40)	49.00 (39.80)	45.00 (35.43)	0.00
TINN, ms	762.33 (154.69)	743.32 (134.09)	747.98 (112.43)	0.00
Maximum NN, ms	798.00 (219.00)	782.00 (164.00)	778.00 (117.50)	0.00
Minimum NN, ms	708.00 (146.00)	692.00 (138.00)	710.00 (83.50)	0.00
MAX‐MIN, ms	72.00 (61.00)	64.00 (64.00)	60.00 (55.00)	0.00
rMSSD, ms	14.16 (15.08)	13.07 (13.31)	11.94 (10.65)	0.00
Triangular index	4.50 (2.60)	4.40 (2.75)	4.05 (2.22)	0.00
AC, ms	−5.20 (6.29)	−5.11 (5.54)	−3.94 (2.98)	0.00
DC, ms	6.36 (7.02)	5.15 (4.94)	4.40 (4.23)	0.00
SD1, ms	10.27 (14.58)	9.50 (12.86)	8.39 (7.91)	0.00
SD2, ms	21.93 (20.50)	20.31 (18.79)	19.75 (15.96)	0.00
pVLF, %	42.84 (31.15)	50.54 (35.31)	60.37 (35.98)	0.00
pLF, %	54.00 (29.64)	46.81 (31.64)	38.06 (35.22)	0.00
DFA alpha1	0.85 (0.40)	0.94 (0.41)	1.01 (0.50)	0.00

Abbreviations: Avg, average; CPC, cardiorespiratory coupling coefficient; I:E ratio, inspiratory to expiratory time ratio; and RMS, root mean square.

*Note*: Inspiratory, expiratory, and tidal volumes represent the cumulative values across each breathing segment recorded at 10, 12, 14, and 16 breaths/min.

**TABLE 6:** Post‐treatment radiological characteristics according to the RICIST v1.1.

Characteristics	PR (*N* = 41)	SD (*N* = 69)	PD (*N* = 10)	*p‐value*
**Size, mm**				
MaxD‐tumor 1	1.80 (2.05)	2.40 (2.6)	0.80 (3.33)	0.06
MaxVD‐tumor 1	1.35 (1.50)	1.80 (1.93)	0.65 (1.65)	0.06
MaxD‐ tumor 2	0.00 (0.00)	0.00 (0.60)	0.00 (0.70)	0.06
MaxVD‐tumor 2	0.00 (0.00)	0.00 (0.40)	0.00 (0.50)	0.01
MaxD‐larger nodule	0.00 (0.00)	0.00 (1.00)	0.00 (0.55)	0.03
MaxVD‐larger nodule	0.00 (0.00)	0.00 (0.70)	0.00 (0.53)	0.02

**TABLE 7:** Post‐treatment biological characteristics according to the RICIST v1.1.

Characteristics	PR (*N* = 41)	SD (*N* = 69)	PD (*N* = 10)	*p‐value*
CEA, ng/mL	2.56 (2.13)	2.91 (3.71)	1.90 (1.51)	0.67
CA15‐3, U/mL	11.97 (8.63)	10.75 (8.69)	8.28 (1.73)	0.66
CA19‐9, U/mL	7.39 (17.13)	11.18 (24.78)	6.42 (0.76)	0.95
NSE, ng/mL	11.35 (5.58)	10.15 (3.72)	9.30 (0.97)	0.11
CA15‐4, U/mL	0.90 (0.50)	0.95 (0.48)	0.88 (0.28)	0.80
Cyfra21‐1, ng/mL	1.83 (1.42)	1.80 (1.41)	1.74 (22.72)	0.94
CA125, U/mL	20.40 (14.85)	17.00 (15.20)	37.00 (20.40)	0.43
ProGRP, pg/mL	45.24 (48.55)	35.17 (20.70)	28.26 (9.18)	0.02

### 3.2 Model evaluation framework

Single‐modal and multi‐modal models were evaluated to predict PR (response) and PD (progression), with key results below.

### 3.3 Addressing class imbalance

To mitigate imbalance (34% PR, 58% SD, 8% PD), 5 non‐overlapping segments were extracted from each PD patient's respiratory recordings, resulting in a total of 200 PD signal segments. Similarly, 164 and 276 segments were retained for the PR and SD groups, respectively, optimizing the ratio to PD:PR:SD = 1:1.64:1.38.

### 3.4 Feature importance

**FIGURE 4:**
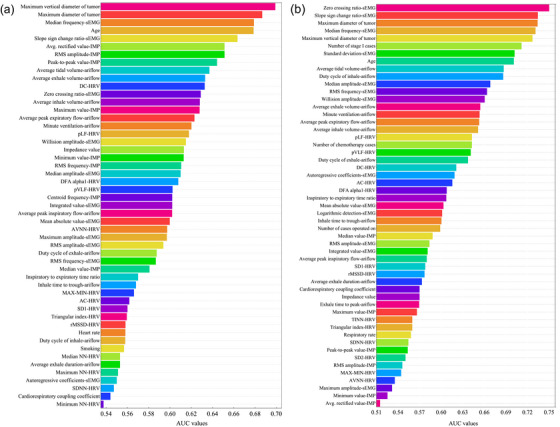
Relative importance of candidate predictors for model development. (a) Top 51 predictors predicting PR and (b) Top 53 predictors predicting PD.

Univariate screening of 58 baseline variables identified the 51 strongest PR predictors (highest AUC = 0.699 for tumour MaxVD; Figure 4a) and the 53 strongest PD predictors (highest AUC = 0.750 for sEMG zero‐crossing ratio; Figure 4b). After applying an AUC ≥ 0.60 threshold, 28 (mostly physiological) predictors were retained for Strategy I and 30 (imaging‐enriched) for Strategy II. The radiation oncologist appended six additional variables—some with slightly lower AUC but deemed clinically relevant—yielding 34 and 36 predictors for multivariate modeling in Strategy I and Strategy II, respectively.

### 3.5 Predictive model performance

**FIGURE 5:**
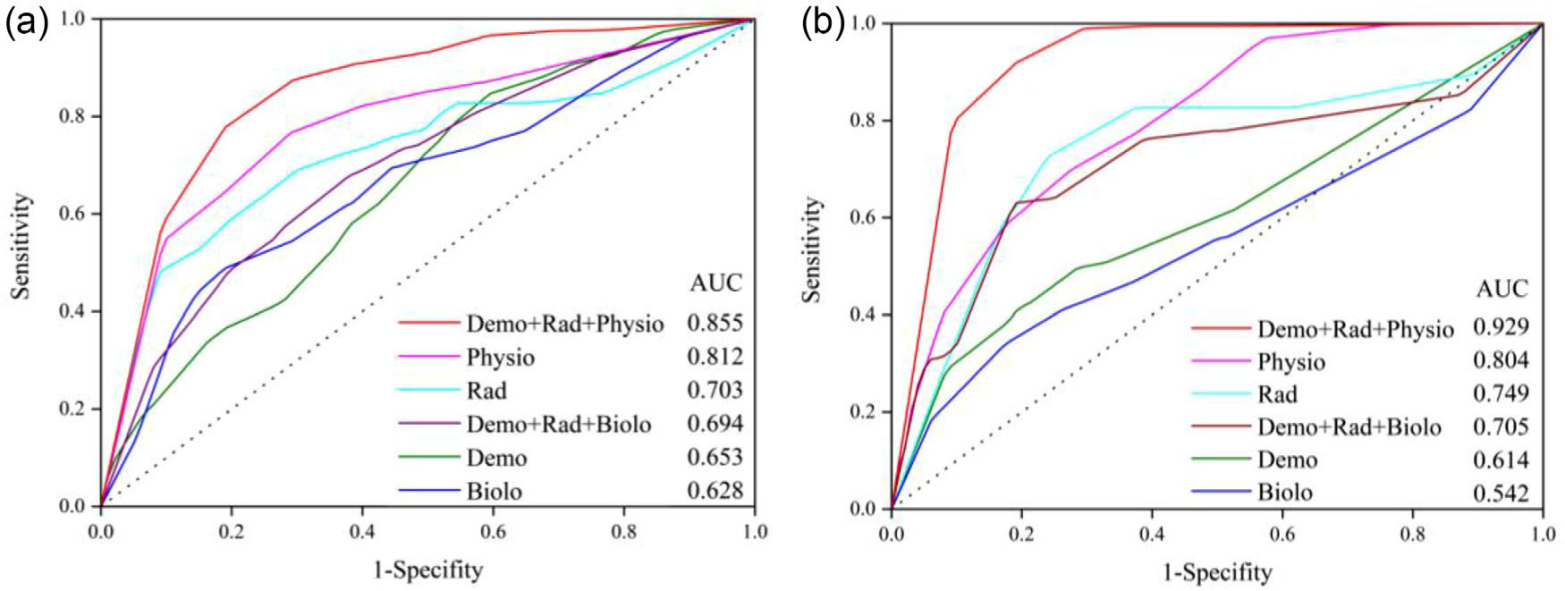
ROC and corresponding AUC value for predicting PR (a) and PD (b).

Models were compared using AUC (primary endpoint) and secondary metrics. ROC curves with corresponding AUC values (Figure 5) and detailed metrics (Tables 8 and 9) were used to evaluate performance. For intuitive visualization of AUC across all modality combinations (single‐modal: Demo, Rad, Biolo, Physio; multi‐modal: Demo+Rad+Biolo, Demo+Rad+Physio) with 95% CIs (error bars), see Figure S1 (PR prediction) and 2 (PD prediction). These charts further confirm the superior performance of the Demo+Rad+Physio model and the variability across configurations.

**TABLE 8:** Performance metrics and 95% confidence interval for predictive models of PR.

**Models**	**Measure**	**AUC**	**Acc**	**Sen**	**Spe**	**PPV**	**F1‐score**
Demo	Mean	0.653	0.639	0.436	0.700	0.210	0.208
	95% CI	[0.516, 0.773]	[0.562, 0.698]	[0.196, 0.733]	[0.609, 0.807]	[0.055, 0.456]	[0.082, 0.337]
Rad	Mean	0.703	0.727	0.690	0.760	0.404	0.461
	95% CI	[0.576, 0.801]	[0.678, 0.795]	[0.550, 0.821]	[0.679, 0.842]	[0.240, 0.585]	[0.357, 0.588]
Biolo	Mean	0.628	0.635	0.554	0.704	0.408	0.427
	95% CI	[0.541, 0.715]	[0.536, 0.715]	[0.351, 0.802]	[0.607, 0.796]	[0.320, 0.481]	[0.367, 0.499]
Physio	Mean	0.812	0.759	0.621	0.825	0.631	0.610
	95% CI	[0.732, 0.890]	[0.696, 0.817]	[0.593, 0.653]	[0.738, 0.910]	[0.436, 0.800]	[0.483, 0.716]
Demo+Rad+Biol	Mean	0.694	0.658	0.541	0.731	0.389	0.395
	95% CI	[0.659, 0.732]	[0.580, 0.735]	[0.411, 0.680]	[0.671, 0.789]	[0.203, 0.575]	[0.270, 0.527]
Demo+Rad+Physio	Mean	0.855	0.815	0.714	0.863	0.723	0.701
	95% CI	[0.843, 0.875]	[0.792, 0.837]	[0.608, 0.792]	[0.813, 0.922]	[0.607, 0.819]	[0.656, 0.744]

Abbreviations: ACC, accuracy; AUC, area under the curve; Biolo, biologic; Demo, Demographic; NPV, negative predictive value; Physio, physiologic; PPV, positive predictive value; Rad, radiologic; Sen, Sensitivity; and Spe, specificity.

**TABLE 9:** Performance metrics and 95% confidence interval for predictive models of PD.

Models	Measure	AUC	Acc	Sen	Spe	PPV	F1‐score
Demo	Mean	0.614	0.648	0.430	0.694	0.219	0.258
	95% CI	[0.514, 0.750]	[0.585, 0.710]	[0.261, 0.592]	[0.645, 0.742]	[0.086, 0.353]	[0.117, 0.397]
Rad	Mean	0.749	0.769	0.453	0.848	0.522	0.449
	95% CI	[0.599, 0.899]	[0.684, 0.856]	[0.238, 0.662]	[0.733, 0.963]	[0.142, 0.901]	[0.132, 0.766]
Biolo	Mean	0.542	0.617	0.291	0.789	0.406	0.323
	95% CI	[0.378, 0.682]	[0.555, 0.683]	[0.108, 0.475]	[0.708, 0.885]	[0.121, 0.691]	[0.121, 0.525]
Physio	Mean	0.818	0.822	0.721	0.831	0.472	0.521
	95% CI	[0.650, 0.976]	[0.742, 0.879]	[0.559, 0.840]	[0.752, 0.894]	[0.160, 0.695]	[0.194, 0.760]
Demo+Rad+Biol	Mean	0.705	0.728	0.511	0.802	0.473	0.465
	95% CI	[0.496, 0.887]	[0.553, 0.887]	[0.203, 0.819]	[0.706, 0.919]	[0.194, 0.784]	[0.179, 0.784]
Demo+Rad+Physio	Mean	0.929	0.885	0.812	0.917	0.791	0.793
	95% CI	[0.899, 0.960]	[0.850, 0.912]	[0.766, 0.864]	[0.869, 0.960]	[0.671, 0.897]	[0.730, 0.841]

#### 3.5.1 PR prediction

The Demo+Rad+Physio multimodal data model demonstrated predictive validity with an AUC of 0.855 (95% CI 0.843–0.875). The model achieved an accuracy, sensitivity, specificity, PPV, and F1‐score of 0.815, 0.714, 0.863, 0.723, and 0.701, respectively.

As shown in Figure S1, physiological single‐modal data (AUC 0.812, 95% CI 0.732–0.890) outperformed biological (AUC 0.628, 95% CI: 0.541–0.715) and demographic (AUC 0.653, 95% CI: 0.516–0.773) single‐modal counterparts. Model excluding physiological data (Demo+Rad+Biolo) showed diminished performance, with an AUC of 0.694 (95% CI 0.659–0.732) and accuracy of 0.658. These results, consistent with the modality‐combination trends in Figure S1, indicate that integrating physiological signals significantly enhances PR prediction compared to models relying solely on demographic, radiological, or biological data.

#### 3.5.2 PD prediction

The Demo+Rad+Physio multimodal data model achieved an AUC of 0.929 (95% CI: 0.900–0.960), with an accuracy, sensitivity, specificity, PPV, and F1‐score of 0.885, 0.812, 0.917, 0.791, and 0.793, respectively.

Figure S2 illustrates that physiological single‐modal data (AUC 0.804, 95% CI: 0.650–0.976) outperformed biological (AUC 0.542, 95% CI: 0.378–0.682) and demographic (AUC 0.614, 95% CI: 0.514–0.750) single‐modal models. Excluding physiological data (Demo+Rad+Biolo) resulted in reduced performance (AUC 0.705, 95% CI: 0.496–0.887). The marked drop in AUC (from 0.929 to 0.705) when excluding physiological data, aligning with Figure S2’s modality‐combination results, highlights their critical role in PD prediction.

### 3.6 Model robustness

To validate the reliability of the optimal Demo+Rad+Physio multimodal model, we assessed its calibration, stability across cross‐validation folds, and generalization ability (i.e., resistance to overfitting).

#### 3.6.1 Calibration

**FIGURE 6:**
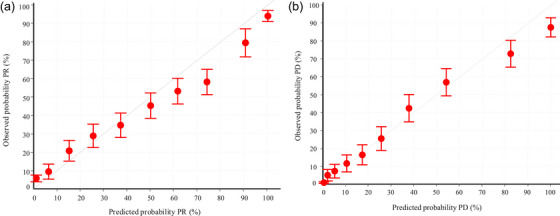
Agreement between predicted and observed probabilities, with a focus on PR (a) and PD (b) cases.

Calibration plots in Figure 6, with 95% CI, show agreement between predicted and observed probabilities for both PR (Figure 6a) and PD (Figure 6b) predictions. Data points closely align with the diagonal line, indicating high calibration accuracy. The concentration of points at lower probabilities suggests the model's predictions are more reliable and accurate in this range. Despite slight overestimation at higher probabilities, the models effectively predict PR and PD, with mean squared errors of 0.07 and 0.01, respectively.

#### 3.6.2 Stability across cross‐validation folds

For the Demo+Rad+Physio multimodal data model: the PR class exhibited an F1‐score with a mean of 0.701 (95% CI: 0.656–0.744), a SD of 0.047, and a CV of 6.721%, alongside an MCC with a mean of 0.538 (95% CI: 0.502–0.568), a SD of 0.049, and a CV of 9.112%; for the PD class, the F1‐score presented a mean of 0.793 (95% CI: 0.730–0.841), a SD of 0.067, and a CV of 8.460%, while the MCC had a mean of 0.731 (95% CI: 0.653–0.799), a SD of 0.091, and a CV of 12.403%.

#### 3.6.3 Overfitting control

Learning curves (Figures S3 and S5) and validation loss curves (Figures S4 and S6) were analyzed to assess overfitting risks in the five‐fold cross‐validation for PR and PD prediction. Across all folds, the training and validation loss curves exhibited a high degree of alignment. For PR prediction (Figure S3), validation losses converged smoothly, showing no signs of overfitting (i.e., no upward trend in later epochs). The five‐fold validation losses stabilized at a low level (around 0.1, Figure S4). For PD prediction (Figure S5), a similar convergence pattern was observed, with the five‐fold validation losses reaching a stable state (Figure S6). The early stopping mechanism—triggered when validation loss stabilized—halted training at optimal epochs, contributing to this stable convergence.

## 4 DISCUSSION

RT is a cornerstone of lung cancer treatment, but variability in patient responses remains a significant clinical challenge. Predicting treatment response could enable timely modifications, aid better treatment decisions, and identify patients unlikely to benefit from RT. Our study presents a novel multimodal framework integrating clinical, radiological, biological, and physiological data to predict RT response. The final models achieved good discriminatory ability, with AUC values of 0.855 for PR and 0.929 for PD.

Lung cancer is a complex and highly heterogeneous disease, with significant differences in clinical characteristics, imaging manifestations, biological behavior, and respiratory physiological parameters among tumors of different patients.^25^, ^26^ Single‐modal data (e.g., imaging, clinical features, or gene expression) often provide limited information, insufficient to fully reflect the patient's condition and treatment response. Integrating multimodal data significantly improves prediction accuracy and robustness compared to single‐modal models, supporting personalized treatment planning by helping clinicians select optimal strategies, thus enhancing treatment effectiveness and reducing unnecessary side effects.^27^ Therefore, our study integrated demographic, radiological, biological, and physiological characteristics to capture disease aspects relevant to RT response.

In this study, lung cancer patients were initially categorized into three groups according to the RECIST: PR, SD, and PD. The National Comprehensive Cancer Network guidelines^28^ recommend re‐evaluation after 4 weeks for patients with SD, while PD patients require immediate intervention. Accurate identification of PR is crucial for tailoring treatment intensity and optimizing resource allocation; similarly, timely identification of PD can prevent further disease progression. Given the poor performance of the three‐category model, we reorganized groups for binary classification based on clinical significance: distinguishing PR from SD+PD (to assess treatment response) and PD from PR+SD (to identify progression risk). This strategy leverages the distinct biological behavior and prognosis of PR and PD (easier for models to capture), while avoiding the confounding effect of SD—highly heterogeneous, including patients with minimal response or slow progression, which complicates separate classification.^29^ By focusing on key clinical issues in stages (response judgment and progression warning), the final models effectively distinguished PR and PD, meeting practical needs for RT response assessment.

To operationalize this strategy for clinical decision‐making, we implemented a two‐stage prediction pipeline to classify new patients into the three original RECIST categories. First, the response model (predicting PR vs. SD+PD) is applied. Patients classified with high probability as PR are assigned to the PR group. The remaining patients (predicted as non‐PR) are then processed by the progression model (predicting PD vs. PR+SD). Those identified as PD form the PD group, while the remainder—now effectively negative for both PR and PD—are classified as SD. This cascading approach leverages the strengths of each binary model to deliver a clinically interpretable, three‐category output.

Radiological imaging is crucial for predicting RT response in lung cancer. Patients with larger initial tumors often experience tumor shrinkage, likely due to RT‐induced death of better‐oxygenated peripheral cells. Conversely, smaller tumors may show stable or progressive disease, possibly due to intrinsic radio‐resistance or aggressive biology.^30^ Modern RT techniques effectively shrink large tumors but struggle with small ones—precision here is crucial to avoid insufficient cell death and progression.^31^ Large tumors may also induce a stronger immune response during RT, aiding regression, while small tumors might not, leading to stabilization or progression.^32^

Our study revealed physiological features ranked second in univariate PR prediction and first in PD identification; moreover, the physiological single‐modal model significantly outperformed demographic, radiological, and biologic models. This suggests physiological monitoring may offer unique insights to enhance traditional response evaluation. Previous research links HRV to survival in cancer patients,^33^ and physiological signals monitoring is valuable in sports training, disease diagnosis, and telemedicine.^34^ sEMG characteristics—reflecting respiratory state and movement patterns—are key factors in predicting RT response^35^: monitoring sEMG during RT improves tumor localization precision, treatment efficacy, and normal tissues protection. These signals reflect diaphragm function (contractile force and fatigue), influencing lung ventilation (often compromised by tumor invasion or tissue damage) and correlating with side effect tolerance and treatment response.^36^, ^37^

Beyond clinical and feature‐level insights, our model stability stemmed from methodological synergy between data augmentation and early stopping—critical for predictive modeling in heterogeneous clinical data. Sample expansion systematically augmented the training dataset by extracting non‐overlapping segments from dynamic respiratory signals, addressing physiological data sparsity in small cohorts. This preserved natural variability, ensuring the model learned contextually robust features rather than idiosyncratic noise. Complementing this, early stopping acted as a targeted safeguard, terminating training when validation loss stabilized (e.g., 5–8 epochs per fold) to avoid overfitting to spurious patterns—vital given high‐dimensional multimodal features. This synergy directly underpinned generalized performance (AUCs 0.855 for PR, 0.929 for PD), balancing data richness and training parsimony to mitigate underfitting and overfitting risks. For clinical translation, this rigor strengthens confidence in reliability across unseen patients, though tradeoffs exist: sample expansion increased computational complexity, and early stopping relied on heuristic thresholds. Future work could refine this via adaptive strategies (e.g., dynamic thresholding) to enhance efficiency and robustness.

Our study has several limitations worth noting. First, data imbalance and limited overall sample size represent key constraints: the number of patients with PD remains small relative to PR and SD cases, and the total cohort size—particularly for PD—limits the model's ability to capture the full spectrum of biological variability underlying treatment responses. Despite our efforts to mitigate this through targeted oversampling of the PD subgroup (via non‐overlapping respiratory signal segmentation), strict patient‐wise partitioning in cross‐validation, and rigorous stability checks, the inherent scarcity of representative PD cases may still restrict generalizability—especially in populations with distinct demographic or clinical profiles.

Second, and more critically, the single‐center design introduces critical constraints on external validity, which merits emphasis. Our patient cohort is derived from a single institution, reflecting its unique demographic characteristics, clinical practice patterns (e.g., specific criteria for adjuvant chemotherapy initiation and multidisciplinary team referral pathways), and patient selection biases (e.g., tertiary care referral patterns that may overrepresent complex cases). These center‐specific features mean our model may inadvertently learn associations tied to local practices rather than universal biological or physiological signals of treatment response. For example, the proportion of patients with comorbidities or disease stages in our cohort may differ substantially from those in community hospitals or centers in other geographic regions, where baseline patient profiles and treatment decision‐making processes vary. Such differences could weaken the model's performance when applied to external populations, even if sample size limitations are addressed. To directly address this single‐center limitation, we plan to proactively initiate collaborations with three geographically and structurally diverse centers: two academic medical centers (serving urban, ethnically diverse populations with complex cases) and one community hospital (serving rural, socioeconomically distinct populations with more typical clinical presentations). This multicenter validation framework is designed to explicitly evaluate how model performance varies across key contextual variables, including: (1) demographic variability; (2) treatment practice differences; and (3) clinical case mixes. By quantifying these contextual effects, we aim to identify whether the model's predictive signals are robust across settings or limited to specific institutional contexts—ultimately refining it for broader clinical utility.

Third, the current analysis relies on pre‐treatment and baseline physiological signals, lacking longitudinal monitoring of respiratory and biological markers throughout RT. This limits our ability to capture dynamic changes in treatment response. Integrating weekly physiological monitoring during treatment, paired with serial blood biomarkers, could enhance the model's temporal resolution for early warning of progression. We plan to implement this longitudinal framework in our expanded cohort, with protocols for standardized signal collection at 0, 2, 4, and 6 weeks post‐RT initiation. These limitations highlight the need for cautious interpretation of our findings, particularly in diverse clinical contexts. However, they also underscore actionable directions to strengthen the clinical utility of our approach—through cohort expansion, multicenter validation, and longitudinal data integration—ultimately advancing the goal of personalized prediction of treatment response.

## 5 CONCLUSION

This study establishes that a multimodal integration of pre‐treatment demographic, radiological, and—notably—physiological characteristics is a powerful predictor of RT response in lung cancer. The developed BPNN models successfully predicted both positive (PR) and negative (PD) outcomes, with physiological features contributing critically to the high accuracy, particularly for PD prediction. This approach offers a promising avenue for optimizing treatment planning by proactively identifying patients at risk of progression, thereby paving the way for more personalized and effective management strategies. Future validation in larger, multicenter cohorts is warranted to confirm its generalizability.

## AUTHOR CONTRIBUTION

*Contributed to the data collection, analysis, and writing*: Xiaojuan Duan. *Contributed to the software design and verification*: Yushun Gong. *Contributed to the hardware design and debugging*: Liang Wei. *Contributed to the clinical guidance*: Lu Chen. *Contributed to hardware maintenance*: Xin Song. *contributed to the conceptualization and design of the study*: Yongqin Li. All authors read and approved the final version of the paper.

## CONFLICT OF INTEREST STATEMENT

The authors declare that they have no known financial interests or personal relationships that could have appeared to influence the work reported in this paper.

## ETHICS STATEMENT

This study was approved by the Medical Ethics Committee of Second Affiliated Hospital of Army Medical University, Chinese People's Liberation Army. (Ref: 2022‐027‐01)

This work was carried out at the Department of Oncology, Second Affiliated Hospital (Xinqiao Hospital) of Army Medical University, whose facilities and resources are gratefully acknowledged.

## CLINICAL TRIAL REGISTRY

This study was registered with the Chinese Clinical Trial Registry. (ChiCTR2300069713)

## Supporting information



Supporting Information

Supporting Information

## Data Availability

The data cannot be made publicly available upon publication due to legal restrictions preventing unrestricted public distribution. The data that support the findings of this study are available upon reasonable request from the authors.
