# Supplementary material for: Response prediction of radiotherapy in lung cancer patients using multimodal data
Source: J Appl Clin Med Phys. 2025 Oct 7;26(10):e70277. doi: 10.1002/acm2.70277 (PMC12504048; doi:10.1002/acm2.70277)
Supplement: Supplementary file 1 — Supporting Information [file ACM2-26-e70277-s002.doc]

| **Characteristics** | **Treatment response** | | | **Disease status** | | |
| --- | --- | --- | --- | --- | --- | --- |
|  | PR (N=41) | SD+PD (N=79) | *p-value* | PR+SD (N=110) | PD (N=10) | *p-value* |
| Male Sex, n(%) | 38 (92.68) | 73 (92.41) | 0.96 | 103 (93.64) | 8 (80.00) | 0.34 |
| Age, yr | 55.20 ± 8.31 | 58.18 ± 7.81 | **0.00** | 56.77 ± 8.16 | 61.40± 5.85 | **0.00** |
| Height, cm | 165.02 ± 6.00 | 165.48 ± 6.46 | 0.71 | 165.49± 6.22 | 163.50± 7.06 | 0.34 |
| Wight, kg | 66.88 ± 9.72 | 65.32 ± 10.86 | 0.44 | 65.85± 9.96 | 65.90± 15.74 | 0.99 |
| BMI, kg/m^2^ | 24.54 ± 3.23 | 23.78 ± 3.27 | 0.23 | 24.00± 3.10 | 24.51± 4.92 | 0.76 |
| Chest, cm | 94.60 ± 6.10 | 94.16 ± 6.77 | 0.73 | 94.32± 6.08 | 94.25± 10.70 | 0.98 |
| Smoking, n(%) | 23 (56.10) | 34 (43.04) | **0.04** | 55 (50.00) | 2 (20.00) | 0.07 |
| Drinking, n(%) | 7 (17.07) | 9 (11.39) | 0.39 | 15 (13.64) | 1 (10.00) | 0.75 |
| **Other completed treatments, n(%)** | | | | | | |
| Surgery | 0 (0.00) | 3 (3.80) | 0.21 | 1 (0.91) | 2 (20.00) | **0.00** |
| Chemotherapy | 39 (95.12) | 74 (93.67) | 0.75 | 106 (96.36) | 7 (70.00) | **0.00** |
| Immunotherapy | 14 (34.15) | 24 (30.38) | 0.68 | 37 (33.64) | 1 (10.00) | 0.13 |
| Targeted therapy | 2 (4.88) | 14 (17.72) | 0.48 | 13(11.82) | 3 (30.00) | 0.11 |
| **Medical condition, n(%)** | | | | | | |
| Other lung disease | 9 (21.95) | 14 (17.72) | 0.58 | 21 (19.09) | 2 (20.00) | 0.94 |
| Cardiac disease | 1 (2.44) | 1 (1.27) | 0.67 | 2 (1.82) | 0 (0.00) | 0.67 |
| Hypertension | 6 (14.63) | 17 (21.52) | 0.42 | 22 (20.00) | 1 (10.00) | 0.55 |
| Diabetes | 2 (4.88) | 6 (7.59) | 0.58 | 7 (6.36) | 1 (10.00) | 0.66 |
| Medication | 8 (19.51) | 21 (26.58) | 0.40 | 14 (12.73) | 3 (30.00) | 0.14 |
| **Tissue type, n(%)** | | | | | | |
| Adenocarcinoma | 8 (19.51) | 17 (21.52) | 0.80 | 22 (20.00) | 3 (30.00) | 0.46 |
| Squamous-cell Ca. | 11 (26.83) | 26 (32.91) | 0.50 | 33 (30.00) | 4 (40.00) | 0.52 |
| Small cell lung Ca. | 21 (51.22) | 31 (39.24) | 0.21 | 49 (44.55) | 3 (30.00) | 0.99 |
| Other | 1 (2.44) | 6 (7.59) | 0.56 | 7 (6.36) | 0 (0.00) | 0.60 |

Supplementary Table 1. Demographic characteristics by treatment response and disease status.

Supplementary Table 2. Baseline radiological characteristics by treatment response and disease status.

| **Characteristics** | **Treatment response** | | | **Disease status** | | |
| --- | --- | --- | --- | --- | --- | --- |
|  | PR (N=41) | SD+PD (N=79) | *p-value* | PR+SD (N=110) | PD (N=10) | *p-value* |
| **Size - mean (SD)** (L, mm) | | | | | | |
| MaxD-tumor 1 | 3.70 (2.20) | 2.50 (2.65) | **0.01** | 3.10 (2.95) | 0.70 (1.23) | **0.00** |
| MaxVD-tumor 1 | 2.60 (2.60) | 1.90 (1.90) | **0.00** | 2.30 (1.88) | 0.40 (1.40) | **0.01** |
| MaxD- tumor 2 | 0.00 (0.00) | 0.00 (0.30) | 0.89 | 0.00 (0.38) | 0.00 (0.00) | 0.06 |
| MaxVD-tumor 2 | 0.00 (0.00) | 0.00 (0.25) | 0.99 | 0.00 (0.30) | 0.00 (0.00) | 0.06 |
| MaxD-larger nodule | 0.00 (0.90) | 0.00 (0.80) | 0.68 | 0.00 (1.00) | 0.40 (0.60) | 0.94 |
| MaxVD-larger nodule | 0.00 (0.80) | 0.00 (0.70) | 0.69 | 0.00 (0.80) | 0.30 (0.53) | 0.90 |
| **Stage - n (%)** | | | | | | |
| Stage Ⅰ | 1 (2.44) | 10 (12.66) | 0.07 | 6 (5.45) | 5 (50.00) | **0.00** |
| Stage Ⅱ | 3 (7.32) | 3 (3.80) | 0.46 | 6 (5.45) | 0 (0.00) | 0.45 |
| Stage III | 28 (68.29) | 39 (49.37) | 0.06 | 64 (58.18) | 3 (30.00) | 0.08 |
| Stage Ⅳ | 8 (19.51) | 26 (32.91) | 0.12 | 32 (29.09) | 2 (20.00) | 0.53 |
| **Location - n (%)** | | | | | | |
| Left upper lobe | 11 (26.83) | 25 (31.65) | 0.59 | 31 (28.18) | 5 (50.00) | 0.15 |
| Left lower lobe | 6 (14.63) | 12 (15.19) | 0.94 | 17 (15.45) | 1 (10.00) | 0.65 |
| Left hilum | 6 (14.63) | 9 (11.39) | 0.61 | 15 (13.64) | 0 (0.00) | 0.22 |
| Right upper lobe | 8 (19.15) | 17 (21.52) | 0.80 | 24 (21.82) | 1 (10.00) | 0.38 |
| Right middle lobe | 3 (7.32) | 6 (7.59) | 0.96 | 9 (8.18) | 0 (0.00) | 0.35 |
| Right lower lobe | 4 (9.76) | 15 (18.99) | 0.19 | 16 (14.55) | 3 (30.00) | 0.20 |
| Right hilum | 4 (9.76) | 8 (10.13) | 0.95 | 12 (10.91) | 0 (0.00) | 0.27 |

MaxD: maximum diameter; MaxVD: maximum vertical diameter

Supplementary Table 3. Baseline biological characteristics by treatment response and disease status.

| **Characteristics** | **Treatment response** | | | **Disease status** | | |
| --- | --- | --- | --- | --- | --- | --- |
|  | PR (N=41) | SD+PD (N=79) | *p-value* | PR+SD (N=110) | PD (N=10) | *p-value* |
| CEA, ng/ml | 2.68 (5.09) | 2.44 (2.82) | 0.31 | 2.58 (3.30) | 2.47 (1.80) | 0.62 |
| CA15-3, U/ml | 12.50 (6.17) | 11.92 (8.14) | 0.63 | 12.30 (7.76) | 12.50 (6.96) | 0.78 |
| CA19-9, U/ml | 8.58 (15.87) | 6.40 (6.86) | 0.48 | 8.67 (16.30) | 5.44 (0.35) | 0.63 |
| NSE, ng/ml | 13.19 (6.80) | 10.32 (3.86) | **0.02** | 11.65 (5.07) | 8.98 (2.57) | **0.05** |
| CA15-4, U/ml | 0.90 (0.83) | 0.80 (0.75) | 0.32 | 0.80 (0.80) | 0.70 (1.50) | 0.53 |
| Cyfra21-1, ng/ml | 2.50 (2.53) | 1.77 (1.06) | 0.55 | 1.86 (1.92) | 1.69 (1.53) | 0.49 |
| CA125, U/ml | 19.00 (23.48) | 17.30 (13.90) | 0.62 | 18.35 (14.23) | 11.40 (7.30) | 0.08 |
| ProGRP, pg/ml | 98.91 (616.28) | 40.04 (32.76) | **0.00** | 50.22 (100.02) | 46.75 (21.61) | 0.29 |

CEA: carcinoembryonic antigen; CA15-3: carbohydrate antigen; CA19-9: carbohydrate antigen; NSE: neuron specific enolase; CA15-4: squamous cell carcinoma antigen; Cyfra21-1: cytokeratin; CA125: carbohydrate antigen; ProGRP: gastrin-releasing peptide precursor.

Supplementary Table 4. Baseline physiologic characteristics by treatment response and disease status.

| **Characteristics** | **Treatment response** | | | **Disease status** | | | |
| --- | --- | --- | --- | --- | --- | --- | --- |
|  | PR (N=41) | SD+PD (N=79) | *p-value* | PR+S (N=110) | PD (N=10) | | *p-value* |
| **Airflow** | | | | | | | |
| Avg inhale volume, L | 65.40 (23.79) | 56.16 (22.30) | 0.00 | 61.60 (23.91) | | 52.32 (21.31) | 0.00 |
| Avg exhale volume, L | 64.14 (22.27) | 55.80 (23.21) | 0.00 | 60.95 (24.77) | | 53.42 (22.33) | 0.00 |
| Avg peak inpiratory flow, L/s | 0.76 (0.23) | 0.68 (0.25) | 0.00 | 0.72 (0.23) | | 0.60 (0.28) | 0.00 |
| Avg peak exspiratory flow, L/s | 0.63 (0.25) | 0.58 (0.20) | 0.00 | 0.60 (0.21) | | 0.56 (0.26) | 0.00 |
| Avg exhale duration, min | 2.55 (0.77) | 2.51 (0.72) | 0.00 | 2.55 (0.71) | | 2.46 (0.77) | 0.00 |
| Avg tidal volume, L | 130.52 (45.45) | 111.35 (42.21) | 0.00 | 123.33 (48.14) | | 104.74 (42.54) | 0.00 |
| Minute Ventilation, L | 28.19 (9.10) | 25.01 (8.14) | 0.00 | 26.81 (8.88) | | 23.17 (8.77) | 0.00 |
| Duty cycle of inhale | 0.43 (0.06) | 0.44 (0.07) | 0.00 | 0.43 (0.06) | | 0.46 (0.10) | 0.00 |
| Duty cycle of exhale | 0.54 (0.07) | 0.53 (0.10) | 0.00 | 0.55 (0.08) | | 0.50 (0.11) | 0.00 |
| Inhale time to trough, s | 0.56 (0.35) | 0.62 (0.37) | 0.01 | 0.62 (0.36) | | 0.56 (0.40) | 0.00 |
| Exhale time to peak, s | 0.82 (0.41) | 0.75 (0.39) | 0.17 | 0.81 (0.38) | | 0.70 (0.43) | 0.04 |
| **Thoracic impedance** | | | | | | | |
| Respiratory rate, bpm | 12.71 (3.71) | 13.34 (4.09) | 0.07 | 12.81 (3.38) | | 13.88 (4.50) | 0.04 |
| I:E ratio | 0.74 (0.21) | 0.76 (0.26) | 0.00 | 0.74 (0.21) | | 0.79 (0.31) | 0.00 |
| Impedance value, Ω | 1.79 (1.39) | 1.33 (0.82) | 0.00 | 1.45 (1.21) | | 1.32 (0.78) | 0.00 |
| Maximum value, Ω | 2.85 (2.03) | 2.05 (1.45) | 0.00 | 2.29 (1.88) | | 2.02 (1.27) | 0.00 |
| Median value, Ω | -0.54 (0.43) | -0.38 (0.35) | 0.00 | -0.38 (0.40) | | -0.50 (0.40) | 0.01 |
| Minimum value, Ω | -2.72 (1.66) | -2.08 (1.03) | 0.00 | -2.19 (1.52) | | -2.18 (0.86) | 0.00 |
| Peak-to-peak value, Ω | 5.35 (3.83) | 4.13 (2.26) | 0.00 | 4.51 (3.38) | | 4.14 (1.84) | 0.00 |
| Avg rectified value, Ω | 1.39 (1.26) | 1.06 (0.54) | 0.00 | 1.10 (1.00) | | 1.12 (0.38) | 0.00 |
| RMS amplitude, Ω | 1.11 (0.03) | 1.11 (0.04) | 0.00 | 1.07 (0.93) | | 0.99 (0.34) | 0.00 |
| Centroid frequency, Hz | 0.31 (0.14) | 0.34 (0.13) | 0.00 | 0.33 (0.15) | | 0.34 (0.09) | 0.42 |
| RMS frequency, Hz | 2.41 (2.46) | 3.06 (1.47) | 0.02 | 2.87 (2.30) | | 2.98 (0.78) | 0.06 |
| **sEMG** | | | | | | | |
| Mean absolute value, µV | 10.42 (7.57) | 9.52 (5.30) | 0.00 | 9.80 (7.20) | | 10.67 (5.07) | 0.00 |
| Standard deviation, µV | 18.81 (13.92) | 17.83 (13.38) | 0.24 | 15.65 (11.50) | | 22.92 (17.84) | 0.00 |
| Maximum amplitude, µV | 112.83 (80.55) | 97.22 (59.81) | 0.00 | 99.16 (70.37) | | 99.36 (48.82) | 0.00 |
| Median amplitude, µV | 0.92 (0.43) | 1.35 (0.86) | 0.00 | 1.35 (0.43) | | 1.35 (0.43) | 0.00 |
| RMS amplitude, µV | 8.11 (5.28) | 7.15 (4.49) | 0.00 | 7.21 (4.96) | | 8.22 (4.34) | 0.00 |
| Zero crossing ratio | 0.14 (0.04) | 0.12 (0.03) | 0.00 | 0.13 (0.04) | | 0.11 (0.02) | 0.00 |
| Slope sign change ratio | 0.29 (0.07) | 0.26 (0.06) | 0.00 | 0.28 (0.07) | | 0.24 (0.06) | 0.00 |
| Willision amplitude | 0.95 (0.04) | 0.94 (0.07) | 0.00 | 0.94 (0.04) | | 0.93 (0.08) | 0.00 |
| Logarithmic detection, µV | 5.21 (3.71) | 5.39 (2.75) | 0.08 | 5.01 (3.48) | | 5.97 (2.20) | 0.01 |
| Integrated value | 10.42 (7.57) | 9.52 (5.57) | 0.00 | 9.80 (7.40) | | 10.72 (5.11) | 0.00 |
| Autoregressive coefficients | -0.15 (0.06) | -0.16 (0.08) | 0.01 | -0.15 (0.07) | | -0.17 (0.11) | 0.00 |
| Median frequency | 29.72 (8.49) | 33.97 (7.99) | 0.00 | 30.72 (7.49) | | 37.22 (8.31) | 0.00 |
| RMS frequency, Hz | 29.98 (16.33) | 32.36 (10.51) | 0.00 | 31.09 (13.66) | | 36.06 (10.69) | 0.00 |
| **HR - HRV** | | | | | | | |
| CPC | 0.56 (0.24) | 0.55 (0.22) | 0.02 | 0.56 (0.23) | | 0.54 (0.24) | 0.00 |
| Heart rate, bpm | 78.77 (18.33) | 81.09 (15.14) | 0.02 | 81.45 (18.23) | | 80.21 (12.38) | 0.24 |
| AVNN, ms | 762.33 (154.69) | 745.40 (128.65) | 0.00 | 746.80 (131.28) | | 747.98 (112.43) | 0.03 |
| Median NN, ms | 760.00 (175.00) | 742.00 (133.00) | 0.01 | 742.00 (166.25) | | 748.00 (113.00) | 0.71 |
| SDNN, ms | 17.13 (13.60) | 15.23 (13.66) | 0.01 | 16.23 (14.73) | | 14.38 (11.10) | 0.00 |
| TINN, ms | 49.00 (40.40) | 46.29 (37.60) | 0.08 | 49.00 (40.17) | | 45.00 (35.43) | 0.00 |
| Maximum NN, ms | 798.00 (219.00) | 782.00 (141.00) | 0.00 | 784.00 (178.50) | | 778.00 (117.50) | 0.65 |
| Minimum NN, ms | 708.00 (146.00) | 698.00 (114.00) | 0.04 | 694.00 (144.50) | | 710.00 (83.50) | 0.63 |
| MAX-MIN, ms | 72.00 (61.00) | 64.00 (60.00) | 0.01 | 68.00 (60.00) | | 60.00 (55.00) | 0.03 |
| rMSSD, ms | 14.16 (15.08) | 12.40 (13.14) | 0.02 | 13.59 (14.75) | | 11.94 (10.65) | 0.00 |
| Triangular index | 4.50 (2.60) | 4.25 (2.60) | 0.00 | 4.45 (2.64) | | 4.05 (2.22) | 0.01 |
| AC, ms | -5.20 (6.29) | -4.54 (4.83) | 0.00 | -5.18 (5.76) | | -3.94 (2.98) | 0.00 |
| DC, ms | 6.36 (7.02) | 4.84 (4.83) | 0.00 | 5.73 (5.65) | | 4.40 (4.23) | 0.00 |
| SD1, ms | 10.27 (14.58) | 8.68 (10.19) | 0.01 | 9.91 (13.95) | | 8.39 (7.91) | 0.00 |
| SD2, ms | 21.93 (20.50) | 19.94 (17.96) | 0.08 | 21.21 (19.49) | | 19.75 (15.96) | 0.03 |
| pVLF, % | 42.84 (31.15) | 55.27 (37.47) | 0.00 | 46.16 (34.41) | | 60.37 (35.98) | 0.00 |
| pLF, % | 54.00 (29.64) | 43.17 (34.71) | 0.00 | 51.19 (31.55) | | 38.06 (35.22) | 0.00 |
| DFA alpha1 | 0.85 (0.40) | 0.96 (0.46) | 0.00 | 0.89 (0.42) | | 1.01 (0.50) | 0.00 |

Avg : average; RMS: root mean square; I:E ratio: Inspiratory to expiratory time ratio; CPC: Cardiorespiratory Coupling Coefficient

Supplementary Table 5. Post-treatment radiological characteristics by treatment response and disease status.

| **Characteristics** | **Treatment response** | | | **Disease status** | | |
| --- | --- | --- | --- | --- | --- | --- |
|  | PR (N=41) | SD+PD (N=79) | *p-value* | PR+SD (N=110) | PD (N=10) | *p-value* |
| **Size - mean (SD)** (L, mm) | | | | | | |
| MaxD-tumor 1 | 1.80 (2.05) | 2.30 (2.90) | 0.09 | 2.20 (2.23) | 0.80 (3.33) | 0.23 |
| MaxVD-tumor 1 | 1.35 (1.50) | 1.70 (1.95) | 0.32 | 1.60 (1.70) | 0.65 (1.65) | 0.09 |
| MaxD- tumor 2 | 0.00 (0.00) | 0.00 (0.60) | 0.79 | 0.00 (0.40) | 0.00 (0.70) | 0.39 |
| MaxVD-tumor 2 | 0.00 (0.00) | 0.00 (0.45) | 0.82 | 0.00 (0.30) | 0.00 (0.50) | 0.34 |
| MaxD-larger nodules | 0.00 (0.00) | 0.00 (0.85) | 0.01 | 0.00 (0.60) | 0.00 (0.55) | 0.86 |
| MaxVD-larger nodules | 0.00 (0.00) | 0.00 (0.70) | 0.01 | 0.00 (0.50) | 0.00 (0.53) | 0.82 |

Supplementary Table 6. Post-treatment biological characteristics by treatment response and disease status.

| **Characteristics** | **Treatment response** | | | **Disease status** | | |
| --- | --- | --- | --- | --- | --- | --- |
|  | PR (N=41) | SD+PD (N=79) | *p-value* | PR+SD (N=110) | PD (N=10) | *p-value* |
| CEA, ng/ml | 2.56 (2.13) | 2.76 (3.13) | 0.79 | 2.65 (3.28) | 1.90 (1.51) | 0.25 |
| CA15-3, U/ml | 11.97 (8.63) | 10.60 (7.96) | 0.99 | 11.50 (9.19) | 8.17 (1.84) | 0.41 |
| CA19-9, U/ml | 7.39 (17.13) | 8.42 (20.32) | 0.43 | 8.55 (23.50) | 9.06 (3.40) | 0.67 |
| NSE, ng/ml | 11.35 (5.58) | 10.06 (3.57) | 0.06 | 10.39 (3.79) | 9.30 (0.97) | 0.20 |
| CA15-4, U/ml | 0.90 (0.50) | 0.95 (0.50) | 0.39 | 0.90 (0.40) | 0.88 (0.28) | 0.76 |
| Cyfra21-1, ng/ml | 1.83 (1.42) | 1.80 (1.45) | 0.37 | 1.83 (1.41) | 1.74 (22.72) | 0.45 |
| CA125:, U/ml | 20.40 (14.85) | 17.05 (21.88) | 0.29 | 17.10 (15.80) | 37.00 (20.40) | 0.82 |
| ProGRP, pg/ml | 45.24 (48.55) | 33.98 (20.22) | 0.01 | 39.79 (25.38) | 28.26 (9.18) | 0.05 |

Supplementary Table 6. All indices of all the signals used in this study

| Signals | Characteristic indices |
| --- | --- |
| AirFlow | Average exhale duration, Average exhale volume, Average inhale duration, Average inhale volume, Average peak expiratory flow, Average peak inspiratory flow, Average tidal volume, Breathing rate, Coefficient of variation of breath volumes, Coefficient of variation of breathing rate, Duty cycle of exhale, Duty cycle of inhale, Minute ventilation, Inhale time to trough, Exhale time to peak |
| sEMG | Mean absolute value, Root mean square, Maximum value, Median value, Crossing zero, Slope sign changes, Willision amplitude, RMSamplitude, Waveform length, Logarithmic detection value, Integrated value, Autoregressive model coefficient, Kurtosi, Skewness, Shape factor, Crest factor, Impulse factor, Clearance factor, Median frequency, Mean Power Frequency, Gravity frequency, Mean square frequency, Root mean square frequency |
| IMP | Maximum value, Minimum value, Mean value, Median value, Peak to peak, Average rectified value, Root mean square, Mean square, RMSamplitude, Kurtosi, Skewness, Shape factor, Crest factor, Impulse factor, Clearance factor, Kurtosis factor, Skewness factor, Gravity frequency, Mean square frequency, Root mean square frequency |
| ECG | Heart rate, AVNN, SDNN, maxRR, minRR, CVRR, MAX-MIN, rMSSD, pNN50, TINN, Triangular index, Total Power, aVLF, aLF, aHF, pVLF, pLF, pHF, nLF, nHF, LF/HF, peakLF, peakHF, ac,dc, SampEn |
