# Supplementary material for: Response prediction of radiotherapy in lung cancer patients using multimodal data
Source: J Appl Clin Med Phys. 2025 Oct 7;26(10):e70277. doi: 10.1002/acm2.70277 (PMC12504048; doi:10.1002/acm2.70277)
Supplement: Supplementary file 2 — Supporting Information [file ACM2-26-e70277-s001.doc]

Supplementary Fig. 1. Impact of Modality Combinations on Predictive PR Model Performance. Error bars represent 95% CI.

Supplementary Fig. 2. Impact of Modality Combinations on Predictive PD. Model Performance. Error bars represent 95% CI.

Supplementary Fig. 3 Learning curves in five - fold cross – validation for predicting PR

Supplementary Fig.4 Losses in five - fold validation cross – validation for predicting PR.

Supplementary Fig. 5 Learning curves in five - fold cross – validation for predicting PD

Supplementary Figure 1. Learning curves in five - fold cross – validation for predicting PD.

Supplementary Fig. 6. Losses in five - fold validation cross – validation for predicting PD.
